# Short-term outcomes of reduced-port laparoscopic surgery versus conventional laparoscopic surgery for total gastrectomy: a single-institute experience

**DOI:** 10.1186/s12893-023-01972-1

**Published:** 2023-03-30

**Authors:** Wenhao Teng, Jingfu Liu, Wenju Liu, Jianping Jiang, Meimei Chen, Weidong Zang

**Affiliations:** 1 Department of Gastrointestinal Surgery, Clinical Oncology School of Fujian Medical University, Fujian Cancer Hospital, Fuzhou, 350014 China; 2 Department of Blood Transfusion, Clinical Oncology School of Fujian Medical University, Fujian Cancer Hospital, Fuzhou, 350014 China

**Keywords:** Reduced-port laparoscopic surgery, Gastric cancer, Laparoscopic surgery, Total gastrectomy, Single-incision plus one port, Single-incision plus two ports

## Abstract

**Background:**

The efficacy of reduced-port laparoscopic surgery (RLS) for total gastrectomy remains unclear. This study focused on evaluating the short-term outcomes of RLS compared with conventional laparoscopic surgery (CLS) for total gastrectomy.

**Methods:**

One hundred and ten patients who underwent completed laparoscopic total gastrectomy for gastric cancer between September 2018 and June 2022 were retrospectively collected and classified into two groups (65 CLS and 45 RLS) according to different operation approach. Twenty-four RLS cases underwent single-incision plus two ports laparoscopic surgery (SILS + 2) and twenty-one underwent single-incision plus one port laparoscopic surgery (SILS + 1). Surgical outcomes, pain intensity, cosmetic and postoperative morbidity, and mortality were compared between groups.

**Results:**

The overall incidence of postoperative complications was similar between the CLS group and the RLS group (16.9% vs. 8.9%, *P* = 0.270). It was also comparable in the Clavien-Dindo classification (*P* = 0.774). However, compared with the CLS group, the RLS group had a significantly shorter total length of incision (5.6 ± 1.0 cm vs. 7.1 ± 0.7 cm, *P* = 0.000); shorter time to first ambulation (24.9 ± 5.9 h vs. 27.6 ± 5.0 h, *P* = 0.009), flatus (3.0 ± 0.8 d vs. 3.5 ± 1.0 d, *P* = 0.022) and oral intake (4.0 ± 1.6 d vs. 6.1 ± 5.1 d, *P* = 0.011); lower white blood cell count on the third day after the operation (9.8 ± 4.0*10^9^/L vs. 11.6 ± 4.7*10^9^/L, *P* = 0.037); and lower visual analogue scale score on postoperative days 1 and 3(3.0 ± 0.7 vs. 3.3 ± 0.7, *P* = 0.044 and 0.6 ± 0.7 vs. 1.6 ± 0.6, *P* = 0.000 respectively). On the other hand, it didn’t find any difference in short-term outcomes between the SILS + 2 group and the SILS + 1 group (*P* > 0.05). But the proximal resection margin was longer in the SILS + 2 group than in the SILS + 1 group (2.6 ± 0.7 cm vs. 1.5 ± 0.9 cm, *P* = 0.046) in patients with adenocarcinoma of the esophagogastric junction (AEG).

**Conclusions:**

RLS for total gastrectomy is a feasible and safe technique when performed by an experienced laparoscopic surgeon. Moreover, compared with SILS + 1, SILS + 2 might have some advantages in AEG patients.

## Introduction

Gastric cancer is the fourth leading cause of cancer-related deaths in the world [1]. It showed comparable outcomes between laparoscopic surgery and open surgery in distal gastric cancer, no matter in early or advanced stage [2–7]. But for total gastrectomy which is more complex, the efficacy of laparoscopic surgery is still being explored. Some studies have demonstrated favorable short-term and long-term results after laparoscopic total gastrectomy by experienced surgeons [8–12].

Omori reported single-incision laparoscopic surgery (SILS) for gastric cancer for the first time in 2011 [13]. It demanded more skills for surgeons to perform this technique because of its difficulty. Therefore, some surgeons preferred to conduct reduce-port laparoscopic surgery (RLS) to optimize the coaxial effect and instrument interference. RLS usually has 1 to 2 additional ports compared with SILS. So we call them single-incision plus one port laparoscopic surgery (SILS + 1) and single-incision plus two ports laparoscopic surgery (SILS + 2). It is not clear which of these two procedures is more advantageous, but they can both reduce the difficulty of operation effectively with minimally invasive. And it could place the drainage tube through the hole of adding port, which seems to be more acceptable instead of no tube or through the incision in the clinic. Recent studies have confirmed the reliable surgical outcomes of RLS in gastric cancer, while most underwent distal gastrectomy [14–16]. Our previous prospective study also demonstrated that it is safe and feasible with less pain to perform SILS + 1 for distal gastric cancer [17]. However, there are few controversial relevant data on total gastric surgery. There is a general agreement that reduced-port laparoscopic gastrectomy is required to be performed in experienced centers, and that young physicians who have not overcome the learning curve need to be trained to perform a variety of single-port laparoscopic training subjects [14, 15, 18]. In this study, we focused on evaluating the short-term outcomes of RLS compared with conventional laparoscopic surgery (CLS) and the potential differences between SILS + 1 and SILS + 2 for total gastrectomy.

## Patients and methods

### Patients

**Fig. 1:**
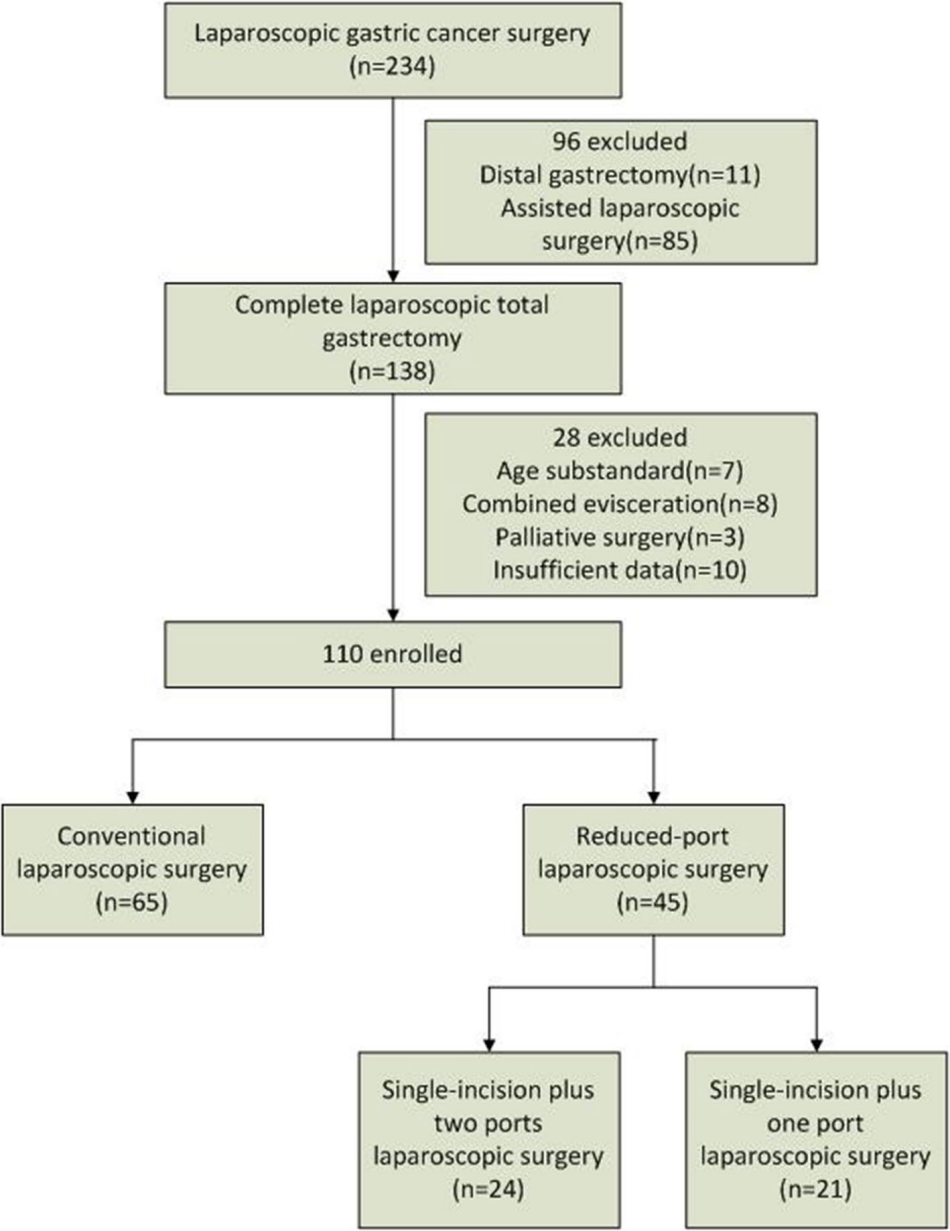
Flow chart of patient selection

Between September 2018 and June 2022, 234 patients with gastric cancer who underwent laparoscopic resection at Fujian Cancer Hospital in China were identified. The age of enrolled patients was from 18 to 75 years. Other inclusion criteria contained the pathological diagnosis of gastric adenocarcinoma with pT1-4aNanyM0 lesions according to the 8th Edition of the AJCC Cancer Staging Manual, and the treatment by complete laparoscopic total gastrectomy. After excluding patients who had either undergone combined evisceration or palliative surgery, and those with insufficient data, 110 patients were finally enrolled in the analysis. According to the approach of operation, they were divided into two groups: CLS (*n* = 65) and RLS (*n* = 45). And there were 24 SILS + 2 and 21 SILS + 1 in the RLS group (Fig. 1). This study was approved by the Medical Ethics Committee of Fujian Cancer Hospital.

### Surgical technique

The surgical position and trocars placement for CLS and SILS + 1 have been described in detail in our previous report [17]. And in the group of SILS + 2, another 5-mm trocar was placed in the right preaxillary line 3 cm below the costal margin based on SILS + 1. All the patients successfully underwent D2 lymphadenectomy according to the lymph node classification established by the Japanese Gastric Cancer Association [18] and Roux-en-Y anastomosis. The resection line of the esophagus was set at least negative margin by intraoperative frozen examination. The intracorporeal esophagojejunostomy was performed with an endoscopic linear or circular stapling device. It conducted functional end-to-end anastomosis (FEEA) [19] in patients using a linear stapling device. While others used a circular stapling device, we processed the following steps. First, the anvil tied with silk was placed into the body through the periumbilical incision. It took a small incision in the esophagus at the level of the resection line, and the anvil was inserted through it and pushed upward above the line. But the end of the silk was outside the esophagus. We used an endoscopic linear stapling device to cut off the esophagus, and then pull out the handle of the anvil with the silk. Second, we removed the specimen through the periumbilical incision and cut off the jejunum at 20 cm from the distal Treitz ligament. Third, here, it used a glove to replace the multiport device. The stapler body and laparoscopy were passed through different fingers of the glove. We inserted the stapler into the distal jejunum about 8 cm and tightened it with a rubber band. Fourth, we placed the stapler and laparoscopy into the body and reconstruct the pneumoperitoneum, and finished the anastomosis. Fifth, after cutting off the rubber band, we move out the stapler and laparoscopy and re-established the multiport device. Finally, it used a linear stapling device to close the jejunum. The jejunojejunostomy was performed using the endoscopic linear stapling device. All procedures were conducted by one experienced surgeon, who had completed more than 500 laparoscopic gastric cancer surgery including at least 50 RLS procedures.

### Postoperative care

**Fig. 2:**
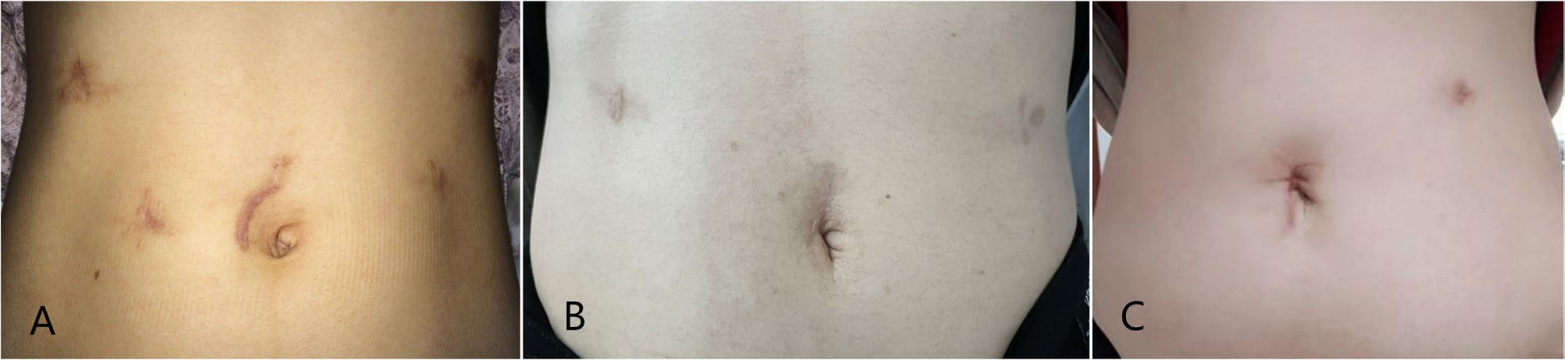
Scars of the incision after the operation. **A** Conventional laparoscopic surgery; **B** Single-incision plus two ports laparoscopic surgery; **C** Single-incision plus one port laparoscopic surgery

All patients were managed by the same surgical team after surgery. Patient-controlled opioid intravenous analgesia (PCIA) was routinely administered immediately after surgery and discontinued 2 days later. It allowed using the additional analgesics if needed. The patients were encouraged to conduct early ambulation and start a liquid diet after exhaustion. The drain was removed at the discretion of the surgeon based on the amount and properties of the drained fluid. When patients were able to tolerate a soft diet and act independently, they were discharged from the hospital. Moreover, we followed up for 30 days after the operation and record the grade of patients’ satisfaction with the cosmetic appearance of the scars (Fig. 2) as low, moderate, or high.

### Statistical analysis

Data were analyzed using SPSS 23.0 and GraphPad Prism 9.2 software. A two-sided *P* < 0.05 was considered significant. For continuous variables, they were expressed by mean ± standard deviation, and an independent t-test or the Mann–Whitney U test was adopted. While categorical variables were presented as numbers and percentages, and the chi-square test or Fisher’s exact test was applied.

## Results

### Patient characteristics

The patients’ clinicopathologic characteristics are presented in Table 1. The baseline characteristics were balanced between the RLS and CLS groups or between the SILS + 2 and SILS + 1 groups (Table 1).

**Table 1:** Baseline clinical characteristics

Characteristics	CLS versus RLS			SILS + 2 versus SILS + 1		
	CLS (*n* = 65)	RLS (*n* = 45)	*P* value	SILS + 2 (*n* = 24)	SILS + 1 (*n* = 21)	*P* value
Age(years)	62.6 ± 8.7	60.0 ± 10.8	0.146	60.0 ± 12.1	60.0 ± 9.4	0.942
Gender			0.778			0.729
Male	52(80.0)	35(77.8)		18(75.0)	17(81.0)	
Female	13(20.0)	10(22.2)		6(25.0)	4(19.0)	
BMI(kg/m^2^)	22.5 ± 3.2	21.6 ± 3.0	0.130	21.8 ± 3.2	21.3 ± 2.7	0.554
ECOG status			0.907			0.051
0	54(83.1)	37(82.2)		17(70.8)	20(95.2)	
1	11(16.9)	8(17.8)		7(29.2)	1(4.8)	
ASA grade			0.905			0.101
I	47(72.3)	33(73.3)		15(62.5)	18(85.7)	
II-III	18(27.7)	12(26.7)		9(37.5)	3(14.3)	
History of abdominal surgery	2(3.1)	4(8.9)	0.224	1(4.2)	3(14.3)	0.326
Staple type for esophagojejunstomy			0.135			0.121
Linear	18(27.7)	7(15.6)		2(8.3)	6(28.6)	
Circular	47(72.3)	38(84.4)		22(91.7)	15(71.4)	
Tumor diameter(cm)	3.9 ± 1.8	3.6 ± 1.9	0.436	3.9 ± 1.8	3.3 ± 2.0	0.284
Tumor location			0.262			0.071
Esophagogastric junction	23	14		11	3	
Gastric fundus	10	3		1	2	
Gastric body	32	28		12	16	
Pathologic TNM stage			0.605			0.134
I	11(16.9)	10(22.2)		4(16.7)	6(28.6)	
II	19(29.2)	15(33.3)		6(25)	9(42.9)	
III	35(53.8)	20(44.4)		14(58.3)	6(28.6)	

*ECOG* Eastern cooperative oncology group, *ASA* American society of anesthesiologists

### Surgical outcomes

As Table 2 shown, no procedure was converted to open surgery in all patients. And there was no statistically significant difference in several surgical outcomes between the CLS and RLS groups, including operation time, blood loss, harvested number of lymph nodes, proximal resection margin, time to first defecation, length of postoperative hospital stays, and cosmetic satisfaction. However, the total length of incision (*P* = 0.000) and time to the first ambulation (*P* = 0.009), flatus (*P* = 0.022), and oral intake (*P* = 0.011) were shorter in the RLS group than in the CLS group. The white blood cell count was lower in the RLS group on the third day after the operation (*P* = 0.037). The visual analogue scale (VAS) score was significantly lower in the RLS group on postoperative days 1 and 3 (*P* = 0.044 and 0.000 respectively). On the other hand, it didn’t find any difference in surgical outcomes between the SILS + 2 group and the SILS + 1 group (*P* > 0.05).

**Table 2:** Surgical outcomes

Surgical outcomes	CLS versus RLS			SILS + 2 versus SILS + 1		
	CLS (*n* = 65)	RLS (*n* = 45)	*P* value	SILS + 2 (*n* = 24)	SILS + 1 (*n* = 21)	*P* value
Operation(min)	196.8 ± 27.6	190.3 ± 27.5	0.231	195.6 ± 16.8	184.3 ± 35.5	0.170
Blood loss(ml)	121.5 ± 41.1	131.1 ± 50.1	0.275	130.0 ± 49.9	132.4 ± 51.5	0.876
Conversion to open surgery	0(0)	0(0)	-	0(0)	0(0)	-
Harvested no. of LN	34.6 ± 14.1	39.6 ± 12.8	0.062	42.3 ± 12.0	36.4 ± 13.3	0.127
Proximal resection margin(cm)	3.6 ± 2.1	3.7 ± 1.9	0.751	3.8 ± 1.8	3.7 ± 2.1	0.838
Positive margin	0(0)	0(0)	-	0(0)	0(0)	-
Total length of incision(cm)	7.1 ± 0.7	5.6 ± 1.0	0.000	5.7 ± 0.9	5.6 ± 1.2	0.812
WBC(*10^9^/L)						
D1	13.6 ± 6.0	11.7 ± 5.1	0.092	11.0 ± 5.3	12.5 ± 5.0	0.359
D3	11.6 ± 4.7	9.8 ± 4.0	0.037	9.0 ± 3.8	10.7 ± 4.1	0.167
Time to first ambulation(h)	27.6 ± 5.0	24.9 ± 5.9	0.009	23.9 ± 4.6	26.0 ± 7.0	0.230
Time to first flatus(days)	3.5 ± 1.0	3.0 ± 0.8	0.022	3.0 ± 0.8	3.1 ± 0.8	0.980
Time to first defecation(days)	4.3 ± 1.2	4.3 ± 1.1	0.959	4.1 ± 1.0	4.5 ± 1.2	0.231
Time to first oral intake(days)	6.1 ± 5.1	4.0 ± 1.6	0.011	3.6 ± 1.3	4.5 ± 1.9	0.080
VAS score						
D1	3.3 ± 0.7	3.0 ± 0.7	0.044	3.2 ± 0.5	2.8 ± 0.8	0.076
D2	2.3 ± 0.5	2.2 ± 0.6	0.455	2.3 ± 0.6	2.1 ± 0.6	0.298
D3	1.6 ± 0.6	0.6 ± 0.7	0.000	0.7 ± 0.8	0.5 ± 0.6	0.362
Additional postoperative analgesics	8(12.3)	4(8.9)	0.758	2(8.3)	2(9.5)	1.000
Length of postoperative hospital stays(days)	12.0 ± 9.2	10.5 ± 4.2	0.299	10.1 ± 2.9	11.0 ± 5.3	0.492
Cosmetic satisfaction			0.570			0.405
Low	2(3.1)	1(2.2)		1(4.2)	0(0)	
Moderate	10(15.4)	4(8.9)		3(12.5)	1(4.8)	
High	53(81.5)	40(88.9)		20(83.3)	20(95.2)	

*LN* Lymph node, *WBC* White blood cell count, *VAS* Visual analogue scale

### Postoperative morbidity and mortality

Postoperative morbidity and mortality are presented in Table 3. No reoperation and mortality occurred in all patients. The overall incidence of postoperative complications was no significant difference between the RLS and CLS groups (8.9% vs. 16.9%, *P* = 0.270) or between the SILS + 2 and SILS + 1 groups (4.2% vs. 14.3%, *P* = 0.326). Moreover, the Clavien-Dindo classification was comparable between them (*P* = 0.774 and *P* = 0.135).

**Table 3:** Postoperative morbidity and mortality

Postoperative morbidity and mortality	CLS versus RLS			SILS + 2 versus SILS + 1		
	CLS (*n* = 65)	RLS (*n* = 45)	*P* value	SILS + 2 (*n* = 24)	SILS + 1 (*n* = 21)	*P* value
Morbidity within 30 days of surgery	11(16.9)	4(8.9)	0.270	1(4.2)	3(14.3)	0.326
Bleeding	0(0)	1(2.2)		1(4.2)	0(0)	
Anastomic leakage	3(4.6)	1(2.2)		0(0)	1(4.8)	
Pulmonary infection	7(10.8)	1(2.2)		0(0)	1(4.8)	
Abdominal infection	1(1.5)	1(2.2)		0(0)	1(4.8)	
Clavien-Dindo classification			0.774			0.135
I	2(3.1)	1(2.2)		0(0)	1(4.8)	
II	4(6.2)	2(4.4)		0(0)	2(9.5)	
III	5(7.7)	1(2.2)		1(4.2)	0(0)	
Reoperation	0(0)	0(0)	-	0(0)	0(0)	-
Re-admission within 30 days of surgery	1(1.5)	0(0)	-	0(0)	0(0)	-
Mortality within 30 days of surgery	0(0)	0(0)	-	0(0)	0(0)	-

### RLS for adenocarcinoma of the esophagogastric junction

**Fig. 3:**
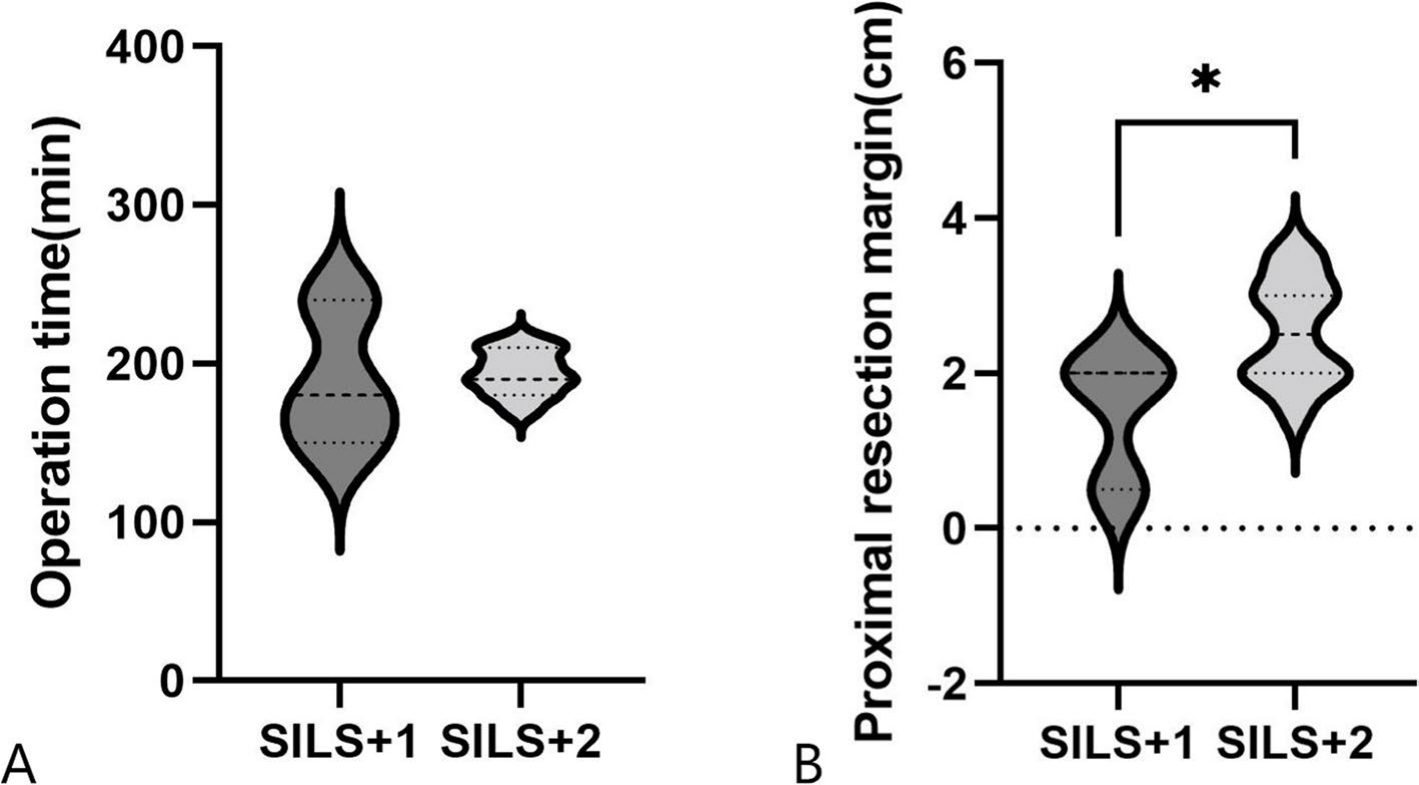
RLS for esophagogastric junction cancer. **A** Operation time in different RLS groups; **B** Proximal resection margin in different RLS groups. *P < 0.05

There were fourteen patients with adenocarcinoma of the esophagogastric junction (AEG) who underwent RLS. The operation time was (190.0 ± 45.8)min and (194.1 ± 14.3)min in the SILS + 1 group and the SILS + 2 group respectively, and there was no statistically significant difference between them (*P* > 0.05). But the proximal resection margin was longer in the SILS + 2 group than in the SILS + 1 group (2.6 ± 0.7 cm vs. 1.5 ± 0.9 cm, respectively; *P* = 0.046) (Fig. 3).

## Discussion

Based on traditional multi-ports laparoscopic surgery, modified reduced-port surgery was developed. Reduced-port laparoscopic gastrectomy requires not only techniques of completely laparoscopic gastrectomy and reconstruction, but also skills of single-port laparoscopic surgery. Total gastrectomy is the most difficult procedure. As a consequence, the effectiveness and safety of performing total gastrectomy by RLS have been controversial. This study, it observed a comparable short-term outcome between RLS and CLS for total gastrectomy. Moreover, RLS has several advantages, including shorter surgical incision, less pain and inflammatory response, and faster postoperative recovery. In addition, there was no significant difference between the SILS + 1 group and the SILS + 2 group. But for patients with esophagogastric junction cancer, it showed a longer proximal margin in the SILS + 2 group. To the best of our knowledge, this study is the first to compare the technique of single-incision plus one port and two ports in patients undergoing laparoscopic total gastrectomy.

Operative time was often utilized as the primary indicator of intraoperative difficulty [20]. Longer operative time means that patients will experience prolonged anesthesia, which not only increases the cost of surgery but also has an impact on morbidity and mortality [21]. RLS reduces the operating ports, which may induce several problems such as instrument conflict and poor exposure. So it is generally considered to increase the difficulty of the operation and needs more time to finish the surgery. Yang et al. [15] conducted a meta-analysis involving 18 studies, and the results showed that the operation time of the RLS group was significantly longer than that of the CLS group. But the studies included in this analysis were all retrospective, and they contained partial gastric banding surgery rather than gastrectomy. Moreover, most of the studies did not specify whether surgeons were proficient in RLS before the trial began. Kunisaki et al. suggest that operation time dropped with surgical experience plateauing after 40 cases for lymph node dissection, and 30 cases for reconstruction in reduce-port laparoscopic total gastrectomy [22]. However, another latest meta-analysis comparing reduced ports surgery and conventional laparoscopic gastrectomy revealed that there was no disadvantage in the operation time of RLS [23]. And it also found the same result in this study. All our enrolled cases were performed by one surgeon who was experienced in RLS, and we believed that some of the differences between RLS and CLS could be overcome with increasing experience. On the other hand, intraoperative blood loss showed similar between the two groups, consistent with other studies [23, 24]. Even some reported less blood loss in RLS [15, 25]. Therefore, the difficulty of total gastrectomy with RLS was acceptable.

Postoperative pain is an important factor affecting recovery. The advantage of RLS with shorten incision is well recognized. But whether it can reduce postoperative pain is still controversial [16, 17, 26, 27]. In our study, the VSA score was used to assess pain in the first 3 days after surgery. It found that the overall score in the RLS group was lower than that in the CLS group, especially on the third day. This might be related to the routine use of PCIA in the first two days after the operation and the increased activity in the later period. Moreover, there should be some relationship between less pain and lower postoperative white blood cell count in RLS patients. In addition, the result revealed that the patients in the RLS group recovered faster after surgery, including early ambulation, early exhaust, and early oral intake. This might be also related to mild postoperative pain. We thought the RLS patients possibly regarded it as a minor operation, which could help them be willing to comply with the treatment and return to normal activities of daily living.

The rate of postoperative complications is an important indicator to evaluate the short-term safety of surgery. Kunisaki et al. [25, 28] found that there was no significant difference in the incidence of complications between reduce-port laparoscopic total gastric surgery and CLS. We got the same result. And it found that none of the patients had serious complications (more than grade IV) evaluated by the Clavien-Dindo classification. On the other hand, oncology efficacy is also a crucial issue that cannot be ignored. To date, only a few studies have reported the long-term outcomes of RLS for total gastrectomy. Kunisaki et al. [25] conducted a propensity score-matched cohort study and found there was no significant difference in 5-year overall survival (91.6% vs. 91.8%, *P* = 0.615) or relapse-free survival (92.3% vs. 92.1%, *P* = 0.587) between the RLS and CLS. Lee et al. [14] compared the long-term oncological outcomes of reduced three-port laparoscopic gastrectomy with a retrospective large-scale multi-institutional study. And it also showed there was no significant difference in 5-year overall survival or disease-free survival between the 3-port and 5-port groups. Although long-term follow-up was not performed in our study, we evaluated the oncology efficacy by the number of lymph nodes harvested [29]. It was said that the extraction of more lymph nodes can improve the staging accuracy and survival outcomes of patients with gastric cancer [30]. In our study, the result showed that the number of lymph nodes retrieved was adequate and did not differ significantly between the two groups. Therefore, reduced-port laparoscopic total gastric surgery is safe in terms of surgical outcomes.

Surgical margin is another key factor to judge the radical resection of the tumor. It declared that the 5-year mortality of patients with positive surgical margins is significantly higher than that of patients with negative surgical margin [31, 32]. In recent years, the incidence of AEG showed an increasing trend [33, 34]. A retrospective study found that the rate of microscopically positive proximal margins in AEG operation was as high as 23.8% [35]. Therefore, adequate proximal margin distance seems to be more important for AEG patients. Mine et al. performed an observational study of 140 patients with AEG and revealed that the long-term outcomes were better in patients with proximal esophageal margin > 2 cm than in those with ≤ 2 cm [36]. In our study, it found that all enrolled AEG patients were pathologically negative after surgery, but the average distance of the proximal margin in the SILS + 2 group was 2.6 cm, which was significantly longer than that in the SILS + 1 group. This may be related to the high proportion of circular staplers to finish the esophagojejunostomy in the SILS + 2 group. When the anastomotic plane is higher than the esophageal fissure, the operation is performed in a narrow thoracic cavity, the view is easily limited using a linear stapler, and the difficulty of closing the common opening is increased when the anastomotic plane is higher. In contrast, using a circular stapler operates with a relatively better view in this situation and it is no need to close the common opening. Therefore, we believe using a circular stapler allows for a higher anastomotic plane and the operators might be more willing to cut more esophageal to guarantee enough margin. Although there was no significant difference in the overall efficacy between the SILS + 2 and SILS + 1 groups, we thought that SILS + 2 might have some advantages in ensuring adequate proximal surgical margin for AEG patients.

Of course, the study has some limitations. It was a retrospective analysis that only assessed the short-term outcomes. More studies on the long-term oncology outcomes of recurrence and survival are planned to perform next. The mean BMI of enrolled patients was 22.12 kg/m^2^, which is not so high. Although 20 patients with BMI > 25 kg/m^2^ were included, given the limited data, it is not yet possible to confirm that it is also safe and feasible to perform RLS in the high BMI population, and it is expected that a separate subgroup analysis will be done for the high BMI population in future studies. Another one is that all procedures were performed by one surgeon, which might ensure better quality control of surgical techniques but also undermine the generality of the results. Therefore, prospective multi-center randomized controlled studies are needed to further evaluate the feasibility of this laparoscopic approach.

## Conclusions

Our results suggest that RLS for total gastrectomy is a feasible and safe technique when performed by an experienced laparoscopic surgeon. Moreover, compared with SILS + 1, SILS + 2 might have some advantages in ensuring adequate proximal surgical margin for AEG patients.

## Data Availability

The datasets used and/or analyzed during the current study are available from the corresponding author.

## Abbreviations

RLS: Reduced-port laparoscopic surgery
CLS: Conventional laparoscopic surgery
SILS + 2: Single-incision plus two ports laparoscopic surgery
SILS + 1: Single-incision plus one port laparoscopic surgery
AEG: Adenocarcinoma of the esophagogastric junction
SILS: Single-incision laparoscopic surgery
FEEA: Functional end-to-end anastomosis
PCIA: Patient-controlled opioid intravenous analgesia
ECOG: Eastern cooperative oncology group
ASA: American society of anesthesiologists
LN: Lymph node
WBC: White blood cell count
VAS: Visual analogue scale
